# Isolation of bacterial extrachromosomal DNA from human dental plaque associated with periodontal disease,using transposonaided capture (TRACA)

**DOI:** 10.1111/j.1574-6941.2011.01166.x

**Published:** 2011-07-29

**Authors:** Philip J Warburton, Elaine Allan, Stephanie Hunter, John Ward, Veronica Booth, William G Wade, Peter Mullany

**Affiliations:** 1 Research Department of Microbial Diseases, UCL Eastman Dental Institute, University College LondonLondon, UK; 2 Research Department of Structural and Molecular Biology, Institute of Structural and Molecular Biology, University College LondonLondon, UK; 3 King's College London Dental Institute, Infection Research Group, Guy's CampusLondon, UK

**Keywords:** oral cavity, plasmid, TRACA, dental plaque, metagenome

## Abstract

The human oral cavity is host to a complex microbial community estimated to comprise > 700 bacterial species, of which at least half are thought to be not yet cultivable *in vitro*. To investigate the plasmids present in this community, we used a transposon-aided capture system, which allowed the isolation of plasmids from human oral supra- and subgingival plaque samples. Thirty-two novel plasmids and a circular molecule that could be an integrase-generated circular intermediate were isolated.

## Introduction

The human oral cavity contains a complex microbial community estimated to comprise > 700 bacterial species (Kazor *et al.*, 2003; Paster *et al.*, 2006; Dewhirst *et al.*, 2010). Mobile genetic elements are a fundamental part of this community, disseminating genes that facilitate adaptation or enabling the exploitation of certain environmental niches. Conjugative transposons have been shown to be responsible for the transfer of antibiotic resistance genes within the oral cavity (Warburton *et al.*, 2007). However, data on the plasmid population within the oral metagenome are limited.

A number of early studies investigating extrachromosomal DNA in bacteria isolated from the oral cavity have reported the presence of small cryptic plasmids in strains of *Streptococcus mutans* (Dunny *et al.*, 1973; Macrina *et al.*, 1977; Caufield *et al.*, 1982; Bergmann *et al.*, 1990). In addition, Yagi *et al.* (1978) reported a plasmid from *Streptococcus sanguis* (now *Streptococcus sanguinis)* that confers resistance to erythromycin, lincomycin and streptogramin. Plasmids have also been identified from a number of other oral bacterial species, including *Bacteroides melaninogenicus* (Vandenbergh *et al.*, 1982), *Prevotella nigrescens* (Teanpaisan *et al.*, 1996), *Fusobacterium nucleatum* (Paula *et al.*, 2003) and the oral treponemes (Ivic *et al.*, 1991; Chan *et al.*, 1996).

However, a major limitation of previous studies investigating the presence of plasmids in oral species is the reliance on bacterial culture, sometimes in conjunction with antibiotic selection. Because approximately 50% of oral bacteria cannot yet be cultured (Wade, 2002; Kazor *et al.*, 2003), plasmids present in these organisms will be missed. Furthermore, plasmids lacking genes conferring resistance to antibiotics will also be missed in studies that use antibiotics as a selection. Therefore, this study uses a transposon-aided capture (TRACA) method (Jones & Marchesi, 2007), which is independent of bacterial culture or phenotype selection, to isolate plasmid DNA from dental plaque samples taken from patients with periodontal disease.

## Materials and methods

### Collection of samples

Supragingival and subgingival plaque samples were collected from 50 patients presenting with periodontal disease ranging from gingivitis through to severe periodontitis, including aggressive periodontitis (UK Research Ethics Committee approval, reference number 06/MRE01/35). All patients were 18 years of age or above, had not taken antibiotics and had not had extensive dental treatment within the previous 3 months. The DNA was extracted from the plaque samples as described by Hunter *et al.* (2011) and pooled.

### TRACA of plasmids

This technique was performed as described previously (Jones & Marchesi, 2007). Briefly, ∼1 μg of metagenomic DNA was digested with plasmid-safe™ DNase (Epicentre) to remove sheared genomic DNA. The sample was then subjected to an *in vitro* transposition reaction using the EZ-Tn5 *Ori*V/Kan2 transposon (Epicentre). The reaction was purified and concentrated to a final volume of ∼10 μL using a YM-100 microconcentrator column (Millipore). All of the EZ-Tn5 reaction was electrotransformed into 100 μL *Escherichia coli* Transformax EPI300-T1^R^ cells (Epicentre) under the following conditions: 18kV cm^−1^, 200 Ω resistance, 25 μF capacitance (Biorad Pulser II) in a prechilled 0.1 cm^3^ electroporation cuvette. Immediately after electroporation, 900 μL of SOC medium was added and the cells were transferred to a 15 mL falcon tube and incubated horizontally at 37°C with shaking at 200 r.p.m., for 1 h. Transformants were selected on Luria–Bertani agar containing 50 μg mL^−1^ kanamycin and incubated at 37°C, aerobically, for up to 48 h.

### DNA sequence analysis

The initial sequence data from each plasmid were obtained using primers FP-1 and RP-1 located at the ends of EZ-Tn5 (Epicentre). The complete sequence of each plasmid was obtained using a primer walking strategy. Given the number of plasmids isolated, a minimum of double sequence coverage was determined. ORFs were defined as nucleotide sequences with the potential to encode proteins > 39 amino acids and preceded by a Shine–Dalgarno sequence at an appropriate distance. Plasmid schematics were constructed using Vector NTI (Invitrogen).

### Nucleotide sequence accession numbers

The DNA sequences of the plasmids have been deposited in GenBank under the following accession numbers: pTRA-CA41 (HM560024), pTRACA42 (HM560025), pTRACA45 (HM560026), pTRACA61 (HM560031), pTRACA63 (HM 560027), pTRACA66 (HM560028), pTRACA69 (HM56 0029), pTRACA73 (HM560030).

## Results and discussion

**Fig. 1:**
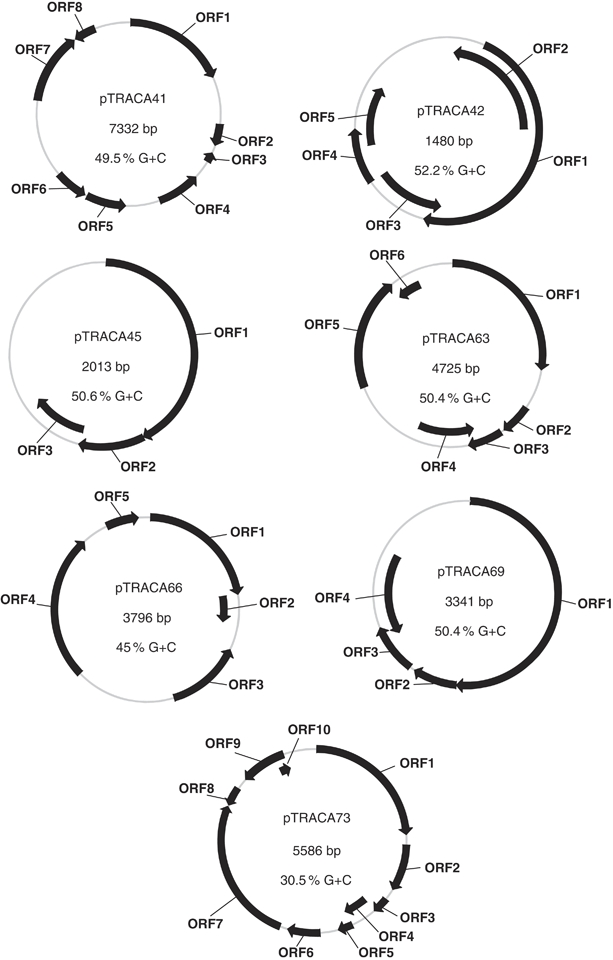
Schematic of the TRACA plasmids showing the putative ORFs, the size and the percentage G+C composition.

DNA from a pooled plaque sample was subjected to a TRACA reaction (see Materials and methods). A total of 33 kanamycin-resistant transformants were isolated, each containing an EZ-Tn5:: plasmid cointegrate. Sequence analysis revealed that the captured circular DNA ranged in size from 0.9 to 7.3 kb, with a G+C range of 30–52% (Fig. 1). Of the 33 plasmids, 29 belong to one of four distinct groups based on their homology to each other (> 92% nucleotide identity): the pTRACA41 group (pTRACA41 and pTRACA58), the pTRACA42 group (pTRACA42, 44, 46, 47, 48, 49, 50, 51, 52, 53, 54, 55, 56, 57, 59, 60, 62, 64, 65, 67, 68, 70 and 72), the pTRACA63 group (pTRACA63 and pTRACA43) and the pTRACA69 group (pTRACA69 and pTRACA71). The remaining four plasmids, pTRACA45, pTRA CA61, pTRACA66 and pTRACA73, share no homology with other plasmids identified in this study.

A number of putative ORFs were identified on each of the plasmids (Fig. 1). The closest matches with the predicted amino acid sequence of these ORFs, identified by blastp analysis, are listed in Table 1. Some of these ORFs are predicted to encode polypeptides with homology to proteins of known function, such as replication, mobilization or plasmid stability. Others encode hypothetical proteins, many of which show no significant homology to sequences in both the NCBI protein and the nucleotide databases, indicating a potential reservoir of genes encoding as yet uncharacterized functions.

**Table 1:** Analysis of the putative ORFs present on the TRACA-isolated plasmids

TRACA plasmid	Putative ORFs	NCBI Protein database top hit	Amino acid identity (overlap)	e-value from blast analysis
pTRACA41 group*	1	Putative Rep protein from pTRACA20, plasmid from uncultured bacterium (YP_003208336)	25% (121/480)	2e-17
	4	Resolvase, *Mitsuokella multacida* (ZP_05404075)	61% (113/184)	2e-57
	7	Integrase family protein, *Desulfurivibrio alkaliphilus* (ZP_05711339)	26% (82/310)	6e-13
	2, 3, 5, 6, 8	No significant homology	–	–
pTRACA42 group†	1	Putative Rep protein from pCL2.1, *Lactococcus lactis* (NP_862690)	43% (84/191)	2e-40
	2, 3, 4, 5	No significant homology	–	–
pTRACA45	1	Putative Rep protein from pJD1, *Neisseria gonorrhoeae* (NP_040411)	71% (201/283)	3e-112
	2	Hypothetical protein, *Neisseria gonorrhoeae* plasmid pJD1 (NP_040412)	86% (65/75)	3e-31
	3	Protein A, *Neisseria gonorrhoeae* plasmid pJD1 (NP_040416)	82% (60/73)	1e-27
pTRACA61	1	Integrase family protein from *Desulfurispirillum indicum* S5 (ZP_06402565)	34% (84/253)	9e-18
pTRACA63 group‡	1	Putative Rep protein from pAB49, *Acinetobacter baumannii* (AAA99423)	27% (93/343)	1e-21
	5	Integrase family protein, *Desulfurivibrio alkaliphilus* (ZP_05711339)	25% (74/294)	3e-8
	2, 3, 4, 6	No significant homology	–	–
pTRACA66	1	Putative Rep protein from pKL001, *Lactococcus lactis* (YP_002332312)	35% (71/200)	1e-27
	4	Integrase family protein, *Desulfurivibrio alkaliphilus* (ZP_05711339)	27% (85/306)	2e-13
	2, 3, 5	No significant homology	–	–
pTRACA69 group§	1	Putative Rep protein from p47L, *Pseudomonas* sp. strain S-47 (YP_232788)	29% (91/305)	7e-22
	2, 3, 4	No significant homology	–	–
pTRACA73	1	Putative Rep protein from pTRACA22, plasmid from uncultured bacterium (YP_003208344)	44% (206/471)	7e-103
	2	Plasmid recombination protein from *Butyrivibrio fibrisolvens* plasmid pRJF2 (YP_002286760)	35% (54/154)	8e-16
	3	Toxin–antitoxin system protein from *Mitsuokella multacida* (ZP_05405007)	57% (41/72)	2e-17
	4	Addiction module toxin, RelE/StbE family *Oribacterium* sp. oral taxon 108 (EGL36808)	69% (61/88)	1e-31
	6	MobC, bacterial mobilization protein from *Clostridium perfringens* (ZP_02863265)	44% (43/98)	3e-10
	7	Relaxase/Mobilization nuclease domain protein *Clostridium* sp. (ZP_06348077)	55% (149/270)	2e-76
	5, 8, 9, 10	No significant homology	–	–

* Includes pTRACA58.

† Includes pTRACA44, 46, 47, 48, 49, 50, 51, 52, 53, 54, 55, 56, 57, 59, 60, 62, 64, 65, 67 68, 70 and 72.

‡ Includes pTRACA43.

§ Includes pTRACA71.

A putative replication (Rep) protein was identified in all except one (pTRACA61) of the plasmids isolated in this study (Table 1). The Rep from pTRACA45 shares 71% amino acid identity to that of pJD1, a 4.2-kb cryptic plasmid from *Neisseria gonorrhoeae* (Korch *et al.*, 1985). Furthermore, the two additional ORFs on pTRACA45 are also closely related to those on pJD1 (Table 1), while its G+C content (50.6%) is similar to that of pJD1 (51.5%) and the genomes of *Neisseria* spp. (∼51%), indicating that it is of neisserial origin.

The Rep proteins of the other 32 plasmids are more distantly related (25–43% amino acid identity) to plasmids found in bacteria belonging to either the *Firmicutes* or the *Proteobacteria* phyla. The pTRACA42-group comprises the majority of the plasmids isolated, 23 in total. This suggests either that this group of plasmids is more abundant in the oral metagenomic DNA and/or is more stable in the *E. coli* host. The plasmids within this group differ in length (1467–1482 bp) and share > 92% nucleotide identity. One plasmid, pTRACA42, was selected for further study. The putative Rep protein is most closely related to that of the small, cryptic plasmid pCL2.1 from *Lactococcus lactis* (Chang *et al.*, 1995) (Table 1). However, the G+C content of the pTRACA42 group of plasmids (∼52%) is considerably higher than that of pCL2.1 (34%) and of *L. lactis* genomes (∼35%), suggesting that they are not of lactococcal origin. The other ORFs on these plasmids have no significant homology to any proteins in the database. Interestingly, nucleotide sequences with over 80% identity to pTRACA42 were identified in one of the two human lung viral metagenomes – project ID: 28439 (Dinsdale *et al.*, 2008). The majority of the sequences in this metagenome were from phage.

The Rep protein from pTRACA66 is also most closely related to that from an *L. lactis* plasmid, specifically pKL001 (Table 1). However, the G+C content of pTRACA66 (45%) is higher than that of pKL001 (32.9%) and the *L. lactis* genomes (∼35%), suggesting that it is not of lactococcal origin. This plasmid contains an ORF with the potential to encode an integrase and three other ORFs that have no significant homology to anything in the protein or the nucleotide databases (Table 1).

The Rep associated with the pTRACA63 group of plasmids are most closely related to that from pAB49, an *Acinetobacter baumannii* plasmid (Table 1). However, the G+C content of pAB49 (38.8%) and the genomes of *Acinetobacter* spp. (38–42%) are much lower than that of pTRACA63 (50.4%), suggesting that pTRACA63 originates from a different bacterial genus. This plasmid also contains an ORF with the potential to encode an integrase.

The Rep proteins associated with plasmids from the pTRACA41 group and pTRACA73 are most closely related to those on pTRACA20 and pTRACA22, respectively, plasmids isolated from the gut metagenome using TRACA (Jones & Marchesi, 2007) (Table 1). Interestingly, the Rep from the pTRACA41 group and pTRACA73 are related to that of pTS1 (24% and 43% amino acid identity, respectively), a cryptic plasmid from the oral bacterium, *Treponema denticola* (Chauhan & Kuramitsu, 2004).

In addition to the *rep* gene, a number of other putative ORFs were identified on each plasmid (Fig. 1.) The identities of the top hits identified by blastp analysis are listed in Table 1. Some of the ORFs on pTRACA73 are predicted to encode polypeptides with shared function, such as mobilization or plasmid stability, to those present on pTRACA22. However, based on sequence analysis, they are only distantly related and the G+C content of pTRACA73 (30.7%) is much lower than of pTRACA22 (51.4%). In contrast, the genes encoding polypeptides on the pTRAC41 group of plasmids share no homology with those present on pTRACA20; however, the G+C content of pTRACA41 (49.5%) is similar to that of pTRACA20 (48.7%).

In contrast to the other 32 plasmids, pTRACA61 does not contain a *rep* gene homologue, but contains an integrase gene homologue sharing 34% amino acid identity to a tyrosine integrase family protein (accession number ZP_06402565). It is possible that this is a circular intermediate of a mobile element. Integrases are site-specific recombinases that frequently produce circular molecules by recombination between their target sites (for reviews, see Smith & Thorpe, 2002; Roberts & Mullany, 2009), providing the intriguing possibility that TRACA has the ability to isolate mobile genetic elements other than plasmids.

The 159 metagenomic data sets currently in the NCBI database were investigated for the presence of the plasmid DNA isolated in this study; however, except for pTRACA41 no homology was found.

This study has identified several novel plasmids, most of which encode hypothetical proteins of unknown function. This shows that there is a relatively unexplored genetic reservoir in the oral metagenome. Although previous studies have reported plasmids in oral streptococci (Dunny *et al.*, 1973; Yagi *et al.*, 1978; Caufield *et al.*, 1982; Vandenbergh *et al.*, 1982), none were captured by the TRACA system. This may be because the bacterial community found at periodontal disease sites is dominated by obligate anaerobes, and streptococci are typically associated with periodontally healthy sites (Paster *et al.*, 2001). However, the *rep* genes associated with pUA140 (Zou *et al.*, 2001) and pLM7 plasmids, from *S. mutans*, could be detected in the sample by PCR amplification (data not shown). Similarly, plasmids previously isolated from gut bacteria were also not captured by Jones & Marchesi (2007), suggesting a limitation of the TRACA system. It is possible such plasmids are unstable in *E. coli*, refractory to transposon insertion or are not present in high enough copy number to enable capture. Furthermore, all the plasmids captured were < 8 kb, mirroring the majority of the previously reported plasmids from oral bacteria, although large plasmids have been reported from the oral cavity (LeBlanc *et al.*, 1993). Whether the isolation of only small plasmids with TRACA is a result of them being numerically dominant in the oral cavity and therefore preferentially captured or a possible limitation of the TRACA system is unknown. It is known that there is a logarithmic decrease in the transformation frequency of plasmids as the size increases; thus, larger plasmids will simply transform less easily into *E. coli* (Szostková & Horáková, 1998). Larger plasmids will also be present in lower copy number, making them harder to capture by TRACA. We are currently investigating whether the substitution of different origins of replication into Tn5 has allowed the capture of different plasmids. It also has to be borne in mind that it is not expected that the TRACA process is likely to capture linear plasmids because the origin of replication used by the modified Tn5 does not have the ability to replicate their extreme termini; these require specialized enzymes (reviewed in Ravin, 2003).

The TRACA protocol has successfully captured novel plasmids from human oral plaque, many of which carry genes encoding as yet uncharacterized functions. TRACA has an advantage over other plasmid isolation techniques as it does not require the expression of plasmid-encoded genes in a surrogate host; thus, as illustrated by this study, novel plasmids and circular molecules can be isolated.

## Statement

Re-use of this article is permitted in accordance with the Term and Conditions set out at http://wileyonlinelibrary.com/onlineopen#OnlineOpen-Terms
